# Aqua walking as an alternative exercise modality during cardiac rehabilitation for coronary artery disease in older patients with lower extremity osteoarthritis

**DOI:** 10.1186/s12872-017-0681-4

**Published:** 2017-09-21

**Authors:** Jong-Young Lee, Kee-Chan Joo, Peter H. Brubaker

**Affiliations:** 1 Division of Cardiology, Department of Internal Medicine, Kangbuk Samsung Hospital, Sungkyunkwan University School of Medicine, Seoul, Republic of Korea; 2 Department of Clinical Exercise Physiology, Seowon University, 377-3 Musimseo-ro, Seowon-gu, Cheongju, Chuncheongbuk 28674 Republic of Korea; 3 Department of Health and Exercise Science, Wake Forest University, Winston-Salem, NC USA

**Keywords:** Exercise, Cardiac, Rehabilitation

## Abstract

**Background:**

The purpose of this study was to examine the effects of aqua walking (AW) on coronary artery disease (CAD) and cardiorespiratory fitness in older adults with osteoarthritis in the lower extremity and compare it with that of traditional over-ground walking.

**Methods:**

Sixty consecutive eligible patients who had undergone percutaneous coronary intervention for CAD with limited ambulation due to lower extremity osteoarthritis were recruited. They were randomly assigned to the AW program group, treadmill/track walking (TW) program group, or non-exercise control group (CON). Assessments were performed before and after 24 weeks of medically supervised exercise training.

**Results:**

Significant differences were observed in the change in %body fat (TW: −2.7%, AW: −2.8%, CON: −0.4%), total cholesterol level (TW: −23.6 mg/dL, AW: −27.2 mg/dL, CON: 15.8 mg/dL), resting heart rate (TW: −6.3 bpm, AW: −6.9 bpm, CON: 1.3 bpm), and cardiorespiratory fitness expressed as VO_2_ peak (TW: 2.3 mL/kg·min^−1^, AW: 2.0 mL/kg·min^−1^, CON: −2.5 mL/kg·min^−1^) over 24 weeks among the groups. However, no significant differences in the change in these measures were found between the TW and AW groups.

**Conclusion:**

AW appears to be a feasible alternative exercise modality to over-ground walking for cardiac rehabilitation and can be recommended for older adults with CAD and osteoarthritis.

## Background

Although primary and secondary prevention programs for coronary artery disease (CAD) are generally multidisciplinary in nature, exercise training is generally considered to be the “cornerstone” intervention. The role of exercise training in the prevention of CAD has been well described [1] and is associated with favorable effects on CAD risk factors such as diabetes mellitus, hypertension, dyslipidemia, and obesity [2–5]. However, many cardiac rehabilitation (CR) program participants cannot exercise sufficiently because of musculoskeletal disorders, including osteoarthritis (OA) [6–8]. This degenerative and often painful musculoskeletal disorder may also limit activities of daily living and leisure time activity. Patients with OA and walking disability are at increased risk of death from cardiovascular causes [9]. Thus, management of OA in patients with CAD should focus on approaches that will improve disability. Cardiovascular rehabilitation programs that increase physical activity have a positive impact on both CAD and OA.

Weight-bearing exercises such as walking are often painful and contraindicated for individuals with OA; thus, non-weight bearing cycling is often recommended [10]. However, this mode of exercise may still be uncomfortable for some patients with OA, and non-weight bearing exercises generally expend fewer calories for the same duration. Consequently, aqua walking (AW) is a potential alternative mode of exercise owing to the potential physiological benefits and no knee pain/irritation [6, 7, 11, 12]. The impact force during walking in the water is well recognized to be lessened owing to buoyancy [7] and to be generally more tolerable than that during over-ground or treadmill/track walking (TW) for patients with OA. Moreover, AW provides a resistance on extremity movements, which results in additive muscular effort and greater metabolic cost. However, the effect of AW and cardiorespiratory fitness (CRF) on CAD has not been thoroughly evaluated. Consequently, the purpose of this study was to determine if AW is a viable alternative mode of exercise for patients with OA who are participating in CAD rehabilitation [6, 7, 13].

## Methods

### Participants

The older adults (aged >65 years) in this study were participants in the CR program performed at Kangbuk Samsung Hospital, which has a therapeutic pool. Consecutive patients with limited ambulation due to lower extremity OA who had undergone percutaneous coronary intervention (PCI) for CAD between October 2015 and September 2016 were enrolled. All the patients with lower extremity OA and persistent pain, which significantly impair their functionality, activity participation, and quality of life, had been treated with pharmacological or non-pharmacological management.

Individualized CR programs were performed 2 to 4 weeks after index PCI. We randomized the patients into three treatment groups. The objective and procedures of the study were explained and understood by each participant prior to signing the informed consent. The participants who performed AW because of arthritic problems of the knee were compared with the participants who performed TW and those in the control group in terms of the change in the CRF after 24 weeks of exercise training. The control group included individuals who did not attend any formal exercise program. Sixty subjects completed the study in one of the three groups as follows: AW (AW group, *n* = 20), TW (TW group, *n* = 21), and control (CON group, *n* = 19). All exercise sessions for both AW and TW were performed in a medically supervised program. This study was approved by the institutional review board of Kangbuk Samsung Hospital. All the participants agreed and permitted participation in this study.

### Laboratory measurements

Prior to performing the laboratory assessments, a questionnaire was administered to each participant to evaluate their medical and family histories, and recent signs/symptoms, and the presence of CAD risk factors for screening purposes. All the participants underwent symptom-limited maximal exercise testing with small increases in workload matched to the subject’s functional capacity every 20 s, which was described in the BSU/Bruce Ramp Protocol. Resting electrocardiography (ECG) findings and blood pressure were measured before starting the test. During the exercise test, the ECG findings were continuously monitored, and a 12-lead rhythm was recorded every 3 min. Systolic (SBP) and diastolic blood pressures (DBP), as well as ratings of perceived exertion, were also obtained every 3 min. Expired gas analysis was performed using a metabolic cart (Moxus, USA) to obtain cardiorespiratory variables, particularly VO_2_ peak. Subjective ratings, including angina, dyspnea, and claudication scale, were queried every 3 min and as needed throughout the test. Immediately after test termination and for every 2 min until the participant fully recovered, the ECG findings, blood pressure, and symptoms were continuously monitored. All exercise tests were symptom-limited and terminated when fatigue and/or shortness of breath occurred. None of the tests were terminated prematurely because of abnormal signs and symptoms, and all participants, including those with OA, achieved an respiratory exchange ratio of ≥1.0 during the exercise test. The VO_2_ peak value was defined as the maximal averaged VO_2_ value attained at end-exercise after the anaerobic threshold was reached.

Blood samples were obtained via the antecubital vein in the fasting state and were analyzed (Hitachi 736–20, Japan) to determine the levels of total cholesterol (TC), low-density lipoprotein-cholesterol (LDL-C), high-density lipoprotein-cholesterol (HDL-C), triglycerides (TGs), and blood glucose (BG). Depression, anxiety, and quality of life were measured using the following questionnaires: Beck Depression Inventory (BDI-II), Beck Anxiety Inventory (BAI), and World Health Organization Quality of Life Questionnaire (WHO-QOL). Percentage of body fat was determined using a bio-impedance analyzer (Biospace, Korea).

### Exercise training program

Both the TW and AW groups participated in medically supervised exercise programs for 24 weeks in a hospital-based rehabilitation setting. Intermittent ECG monitoring, and blood pressure and BG measurement were performed by qualified staff before, during, and after the exercise sessions. The TW group performed either treadmill or track walking of their own choice. The participants in the AW program performed AW and aquatic calisthenics in the therapeutic pool. The depth of submersion was approximately at the height of the xiphoid process of the subjects. The temperature of the water ranged from 30 °C to 32 °C. The heart rate during both AW and TW was continuously monitored using a water-proof heart rate monitor (Polar, Finland). Blood pressure and ECG findings were intermittently measured during both TW and AW by using a sphygmomanometer and defibrillator paddles, respectively.

The intensity of exercise training for the 24-week intervention ranged from 50% heart rate reserve (HRR) to 65% HRR (obtained from the baseline exercise test) in the TW group. In the AW group, the target heart rate was modified by subtracting 15–17 bpm from the 50% HRR to the 65% HRR calculated range, as the heart rate is known to be decreased during water immersion owing to an increased stroke volume [13]. Furthermore, we employed the Borg scale of the rated perceived exertion (RPE) during exercise and used the range of RPE values of 11 to 14 for every exercise session. The exercise time was maintained at 30 min per session for both TW and AW, not including the warm-up and cool-down periods. The exercise frequency was 3 days per week.

### Statistical analyses

All variables were presented as mean ± SD, and a *p* value of .05 was considered statistically significant. The Kolmogorov-Smirnov test was used to examine the normal distribution of each variable. All baseline variables in this study were found to be normally distributed. For all the variables of interest, a two-way repeated-measures analysis of variance was performed to determine any time and/or group main effects, and an interaction (time × group) effect in the three groups from baseline to the 24th week of follow-up. Scheffe post hoc testing was performed on significant effects to determine between- and within-group differences.

## Results

### Baseline characteristics

Table 1 shows the baseline characteristics of all the subjects who were randomly assigned to the treatment groups. The mean age of the subjects was 72.9 ± 4.7 years, with a male sex dominancy (76.6%). Among the treatment groups, no significant differences were found. The exercise programs were performed using the prespecified exercise protocol.

**Table 1:** Demographics and Characteristics

	TW (*n* = 21)	AW (n = 20)	Con (n = 19)
Age (yr)	72.2 ± 4.5	73.2 ± 4.3	72.9 ± 4.7
Height (cm)	159.9 ± 9.6	154.6 ± 7.4	153.7 ± 7.1
Weight (kg)	58.4 ± 10.2	58.7 ± 6.1	57.0 ± 7.7
Gender			
Male	15 (71.4)	14 (70.0)	14 (73.7)
Female	6 (28.6)	6 (30.0)	5 (26.3)
Clinical Status			
Stable angina	11 (52.4)	10 (50.0)	10 (52.6)
Unstable angina	5 (28.7)	5 (25.0)	5 (26.3)
Acute Myocardial infarction	5 (28.7)	5 (25.0)	4 (21.1)
Left ventricle ejection fraction (%)	61.4 ± 6.3	60.9 ± 5.1	61.3 ± 69
Hypertension	11 (52.3)	10 (50.0)	10 (52.6)
Hyperlipidemia	10 (47.6)	9 (45.0)	9 (47.3)
Current smoker	6 (28.6)	5 (25.0)	5 (26.3)
Diabetes mellitus	6 (28.5)	6 (30.0)	6 (31.6)
Medication			
Antiplatelet agent	21 (100)	20 (100)	19 (100)
Beta blocker	16 (76.1)	17 (75.0)	15 (78.9)
ACEI/ARB	10 (47.6)	11 (55.0)	10 (52.6)
Diuretics	3 (14.3)	2 (10.0)	1 (5.0)
Anti-diabetic	2 (9.5)	2 (10.0)	4 (21.0)
Statin	21 (100.0)	20 (100.0)	19 (100.0)

Values are mean ± SD or n (%)

*TW* treadmill/track-walking, *AW* aqua-walking, *Con* control, *CAD* coronary artery disease, *ACEI* angiotensin converting enzyme inhibitor, *ARB* angiotensin receptor blocker, *NSAID* non-steroidal anti-inflammatory drug

### Body composition

**Fig. 1:**
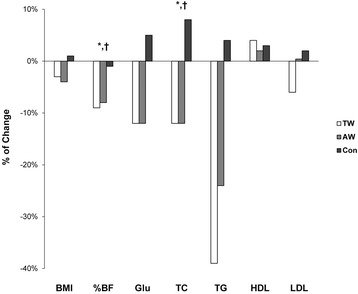
Comparison of the change in body composition, blood lipid level, and blood glucose level. *Difference between the treadmill/track walking (TW) and control (CON) groups. ^**†**^Difference between the aqua walking (AW) and CON groups

Body weight was not statistically different among the groups; no time and group interaction was observed for body weight. Although the time effect and interaction for body mass index (BMI) was significant, the between-group effects were not; the BMI decreased in both the TW and AW groups, and increased in the CON group. The %body fat was significantly different among and within the groups (TW: −2.7%, AW: −2.8%, CON: −0.4%). A significant group and time interaction was also found. The post hoc testing revealed that both the TW and AW groups had significant reductions in %body fat compared with the CON group, although no significant difference was found between the TW and AW groups (Fig. 1).

### CRF and resting Hemodynamics

The peak oxygen consumption increased in both the TW and AW groups after exercise training (+2.3 mL/kg·min^−1^ vs. +2.0 mL/kg·min^−1^ respectively); the CON group demonstrated a decreased peak oxygen consumption (−2.5 mL/kg·min^−1^) after 24 weeks. The post hoc testing revealed that both the TW and AW groups had a significant increase in VO_2_ peak when compared with the CON group; however, no significant difference was observed between the two exercise groups.

**Fig. 2:**
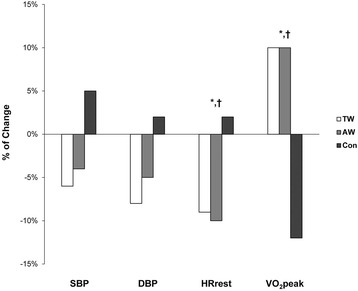
Comparison of the change in hemodynamics and functional capacity. *Difference between the treadmill/track walking (TW) and control (CON) groups. ^**†**^Difference between the aqua walking (AW) and CON groups

Although not statistically significant, SBP decreased in both the TW and AW groups and increased in the CON group. Similarly, no significant main effects or interaction for DBP was found. The resting heart rate was significantly different within the groups, demonstrating a decrease in both the TW and AW groups (−6.3 and −6.9 bpm, respectively). A small change in the resting heart rate in the CON group (1.3 bpm) and a significant interaction of time and group were observed (Fig. 2).

### Fasting blood lipids and BG

Although the mean fasting BG level in both the TW and AW groups decreased after exercise training and that in the CON group increased, no statistically significant difference between and within the groups, and no interaction of time and group were found. The TC level had statistically significant between- and within-group differences (TW: −23.6 mg/dL, AW: −27.2 mg/dL, CON: 15.8 mg/dL) and a significant interaction between time and group. The TC level in both the TW and AW groups decreased, and that in the CON group increased. The post hoc testing revealed that the TC levels of both the TW and AW groups improved significantly when compared with that of the CON group. The TG level was significantly different within the groups, and remarkable decreases were observed in both the TW and AW groups (−63.8 mg/dL and −40.6 mg/dL, respectively) with a slight increase in the CON group (5.6 mg/dL). However, despite these differences, no significant differences among the groups or in the interactions for the TG level were found. No significant main effects or interaction was observed for the LDL-C and HDL-C levels (Fig. 1).

### Psychosocial factors

None of the psychosocial factors, including the BDI-II, BAI, and WHO-QOL findings, had any significant difference within or between the groups, and no significant interaction of time and group was found. It is interesting that the mean depression and anxiety scores decreased in all the groups, while the WHO-QOL scores increased, despite their non-significance.

## Discussion

The present study demonstrated meaningful changes in both the TW and AW groups for %body fat (−9% and −8%, respectively) and BMI (−3% and −4%, respectively) after 24 weeks of training in the older patients with CAD. Given that every patient with OA and CAD could not receive traditional TW programs during their CR program, AW could be a feasible alternative modality.

The levels of exercise and/or physical activity have been well recognized to be inversely associated with the risk of cardiovascular disease. However, traditional weight-bearing exercises such as walking often cannot be tolerated by individuals with orthopedic limitations. Several studies have suggested that water-based exercises are a viable alternative to land-based exercises for individuals with orthopedic problems and excess adiposity [6, 7]. However, this study demonstrated the beneficial effects of the AW program in older individuals who underwent PCI. Daily activities, particularly for mobility-impaired adults, rarely require maximal efforts. Therefore, the ability to perform prolonged submaximal exercises is often more relevant to health-related fitness. From this perspective, the effects of the AW program proposed in this study could be more emphasized by considering submaximal walking training.

Our results are consistent with the changes in the body composition that were reported in most previous studies on water-based exercise therapy [8, 11, 12, 14]. Some studies stated that the effect of body fat reduction was not significant in water-based exercise training because of a tendency of conserving fat to provide warmth and buoyancy during water exercise; however, these statements were limited by the lack of scientific evidence [12]. The changes in body weight and body composition would be similar in these two modes of exercise training, as oxygen consumption and caloric expenditure have been found to be similar between water- and land-based exercises [7]. During water-based walking, water not only increases buoyancy, which reduces energy expenditure, but also provides a resistance against extremity movements, requiring more energy expenditure than the resistance provided by air [15].

While water buoyancy decreases joint forces, which is beneficial to those with discomfort from lower extremity arthritis, the increased resistance also helps strengthen the muscles of the lower extremity and thus improves physical function. CRF, a measure of physical function, is an independent risk factor of cardiovascular and all-cause mortality [16] and even small improvements in this measure are associated with a decreased mortality [17]. Water-based exercise training programs have been shown to increase CRF or maximum exercise time/workload in patients with brain injury [8] and stroke [18], as well as in unfit young individuals [19] and healthy elderly women [20]. In the present study, both the TW and AW groups demonstrated significant and meaningful improvements (10%) in CRF. Conversely, individuals who undergo traditional exercise-based rehabilitation programs commonly have a greater increase (i.e., 15–20%) in CRF levels [21, 22]. This outcome is often based on estimated MET levels and not from directly measured VO_2_ peak levels. The former approach is known to usually overestimate the actual functional capacity, particularly in patients with cardiac conditions [23]. Furthermore, the participants in the present study were older patients (aged >65 years) with lower extremity arthritis and thus are likely to generate smaller improvements in CRF than younger, healthier subjects. The 10% increase in the VO_2_ peak value in both the AW and TW groups in the present study is consistent with the result of a prior study of a water-based exercise program in healthy elderly subjects [20] and still represents a meaningful change in the physical function for these patients. The clinical meaning of the VO_2_ peak value improvement could be assumed. The VO_2_ peak value improvement obtained in the 24-week program may be relevant, not only because it is consistent with the results of a prior study but also because a similar improvement predicted the long-term prognosis of patients with CAD [24, 25]. Our study might emphasize the potential clinical meaning of the AW program in CR programs.

Water-based exercise therapies have the potential to impact hemodynamic measures, including heart rate and blood pressure. A number of studies have concluded that water immersion redistributes blood volume and increases cardiac output because of the hydro-pressure acting on the peripheral vasculature [26–30]. Consequently, heart rate decreases and stroke volume increases at rest and during exercise during water immersion. Furthermore, the increased central blood volume affects the renal blood flow and the right atrial filling pressure, suppressing the arginine vasopressin and plasma renin activity, and activating atrial natriuretic factors. These hormones induce natriuresis and diuresis, which in turn decrease blood pressure [26–28, 31]. Figure 2 shows that SBP and DBP decreased in both the TW and AW groups without any significant difference between them (−6% and −4% in SBP, and −8% and −5% in DBP, respectively). These reductions in blood pressure are meaningful and consistent with those of other exercise training studies [3, 32]. Whether the observed blood pressure reductions are due to the “normal” exercise training effect and/or from the aforementioned mechanism of activating natriuresis and diuresis in water-based exercises remains to be determined [26–28, 31]. The resting heart rate decrease observed in both the TW and AW groups is an expected adaptation to aerobic exercise related to the increased parasympathetic activation [33] and appears to be similar between the two groups.

Several studies have investigated the effects of water-based exercises on the improvement of blood lipids and reported inconsistent results [11, 34, 35]. In the present study, meaningful changes in TC and TG levels were observed in both the TW and AW groups; however, no significant change in HDL-C and LDL-C levels were observed in either groups. While many exercise studies have reported an increase in HDL-C level, the volume of exercise in the present study may have been insufficient to generate this result. The TW and AW interventions in the present study were only performed 30 min three times per week, whereas many studies [36] have used longer duration and/or greater frequency of exercises. We limited the exercise volume of our subjects owing to concerns of worsening their arthritic condition.

Improved insulin sensitivity and glucose control secondary to aerobic exercise training have also been well established [37]. However, only a few studies have examined the effect of water-based exercises on glucose tolerance and/or insulin sensitivity. Those studies reported a significant reduction in fasting plasma glucose level [35] and significant decreases in fasting plasma insulin level, 2-h postprandial insulin/glucose level, and homeostasis model assessment of insulin resistance after 12 weeks of [38] water-based exercise training programs. In the present study, the fasting BG level decreased in both the TW and AW groups (−12% both). Although the differences did not reach any statistical significance, these results suggest that AW has favorable effects on glycemic control that are similar to that of TW.

## Limitations

Our study has several limitations. This study was limited by its small sample size, which may have decreased the occurrence of statistical significances in some measures such as psychosocial measures, although we still observed many meaningful changes in the CAD risk factors and CRF from AW. Furthermore, the lower total exercise “volume” employed in the present study (30 min, three times per week) may have reduced the magnitude of change in some of our outcome measures; however, this was used to prevent worsening of symptoms in the patients with arthritis. Our findings could be difficult to generalize to all patients because performing AW needs specialized facility and members. Our study did not accurately evaluate the level of physical activity out of the hospital, which might have influenced the outcomes of this study. However, our study can provide evidence to support the benefits of exercise training, especially alternative modalities such as AW, in an underrepresented category of patients, including older adults with CAD and OA.

Although maximal cardiopulmonary exercise tests are the gold standard for CRF assessment, they have been described as impractical for evaluating older and mobility-impaired adults [39]. Thus, several alternative submaximal walking protocols have been developed to estimate CRF. However, we did not assess the walking capacity using other walking tests. Thus, the CRF might have been overestimated or underestimated in our study. Improvements in walking distance or pace have been demonstrated as strong independent predictors and a better guide in assessing the prognosis than the gains in the VO_2_ peak value. However, our study did not evaluate these variables. The main aim of our study was to evaluate the feasibility of AW as compared with that of the TW program. Finally, our study did not investigate the clinical outcome of the enrolled patients.

## Conclusions

The results of this study indicate that AW for only 30 min three times per week can elicit the same beneficial effects as TW for the same duration/frequency on body composition, CRF level, blood lipid levels, and BG level, as well as resting hemodynamics, in older patients with CAD who underwent PCI. Consequently, this mode of exercise should be recommended as part of a comprehensive exercise-based prevention/rehabilitation program for patients with CAD who are limited by lower extremity OA.

## Abbreviations

AW: Aqua walking
BAI: Beck anxiety inventory
BDI-II: Beck depression inventory
BG: Blood glucose
CAD: Coronary artery disease
CON: Control
CR: Cardiac rehabilitation
CRF: Cardiorespiratory fitness
DBP: Diastolic blood pressure
HDL-C: High-density lipoprotein cholesterol
HRR: Heart rate reserve
LDL-C: Low-density lipoprotein cholesterol
OA: Osteoarthritis
PCI: Percutaneous coronary intervention
RPE: Rated perceived exertion
SBP: Systolic blood pressure
TC: Total cholesterol
TG: Triglyceride
TW: Treadmill/track walking
WHO-QOL: World Health Organization Quality of Life

## References

[1] Blair SN, Kohl HW, Barlow CE, Paffenbarger RS, Gibbons LW, Macera CA (1995). Changes in physical fitness and all-cause mortality. A prospective study of healthy and unhealthy men. JAMA.

[2] Eriksson JG (1999). Exercise and the treatment of type 2 diabetes mellitus. An update. Sports medicine (Auckland, NZ).

[3] Kokkinos PF, Papademetriou V (2000). Exercise and hypertension. Coron Artery Dis.

[4] Larry Durstine J, Thompson PD (2001). Exercise in the treatment of lipid disorders. Cardiol Clin.

[5] Klein S, Peters EJ, Shangraw RE, Wolfe RR (1991). Lipolytic response to metabolic stress in critically ill patients. Crit Care Med.

[6] Meredith-Jones K, Waters D, Legge M, Jones L (2011). Upright water-based exercise to improve cardiovascular and metabolic health: a qualitative review. Complementary therapies in medicine.

[7] Cassady SL, Nielsen DH (1992). Cardiorespiratory responses of healthy subjects to calisthenics performed on land versus in water. Phys Ther.

[8] Driver S, O'Connor J, Lox C, Rees K (2004). Evaluation of an aquatics programme on fitness parameters of individuals with a brain injury. Brain Inj.

[9] Nuesch E, Dieppe P, Reichenbach S, Williams S, Iff S, Juni P (2011). All cause and disease specific mortality in patients with knee or hip osteoarthritis: population based cohort study. BMJ (Clinical research ed).

[10] Ettinger WH, Burns R, Messier SP, Applegate W, Rejeski WJ, Morgan T, Shumaker S, Berry MJ, O'Toole M, Monu J (1997). A randomized trial comparing aerobic exercise and resistance exercise with a health education program in older adults with knee osteoarthritis. The fitness arthritis and seniors trial (FAST). JAMA.

[11] Volaklis KA, Spassis AT, Tokmakidis SP (2007). Am Heart J.

[12] Gappmaier E, Lake W, Nelson AG, Fisher AG (2006). Aerobic exercise in water versus walking on land: effects on indices of fat reduction and weight loss of obese women. The Journal of sports medicine and physical fitness.

[13] Gulick DT, Brody LT, Geigle PR (2009). Specialized aquatic cardiovascular training. Aquatic exercise for rehabilitation and training.

[14] Saavedra JM, De La Cruz E, Escalante Y, Rodriguez FA (2007). Influence of a medium-impact aquaerobic program on health-related quality of life and fitness level in healthy adult females. The Journal of sports medicine and physical fitness.

[15] Brubaker P, Ozemek C, Gonzalez A, Wiley S, Collins G (2011). Cardiorespiratory responses during underwater and land treadmill exercise in college athletes. J Sport Rehabil.

[16] Blair SN, Brodney S (1999). Effects of physical inactivity and obesity on morbidity and mortality: current evidence and research issues. Med Sci Sports Exerc.

[17] Albright A, Franz M, Hornsby G, Kriska A, Marrero D, Ullrich I, Verity LS (2000). American College of Sports Medicine position stand. Exercise and type 2 diabetes. Med Sci Sports Exerc.

[18] Chu KS, Eng JJ, Dawson AS, Harris JE, Ozkaplan A, Gylfadottir S (2004). Water-based exercise for cardiovascular fitness in people with chronic stroke: a randomized controlled trial. Arch Phys Med Rehabil.

[19] Davidson K, McNaughton L (2000). Deep water running training and road running training improve Vo2 max in untrained women. J Strength Cond Res.

[20] Broman G, Quintana M, Lindberg T, Jansson E, Kaijser L (2006). High intensity deep water training can improve aerobic power in elderly women. Eur J Appl Physiol.

[21] Dugmore LD, Tipson RJ, Phillips MH, Flint EJ, Stentiford NH, Bone MF, Littler WA (1999). Changes in cardiorespiratory fitness, psychological wellbeing, quality of life, and vocational status following a 12 month cardiac exercise rehabilitation programme. Heart.

[22] LaHaye SA, Lacombe SP, Koppikar S, Lun G, Parsons TL, Hopkins-Rosseel D (2014). High and low contact frequency cardiac rehabilitation programmes elicit similar improvements in cardiorespiratory fitness and cardiovascular risk factors. Eur J Prev Cardiol.

[23] Koutlianos N, Dimitros E, Metaxas T, Cansiz M, Deligiannis A, Kouidi E (2013). Indirect estimation of VO2max in athletes by ACSM's equation: valid or not?. Hippokratia.

[24] Kavanagh T, Hamm LF, Beyene J, Mertens DJ, Kennedy J, Campbell R, Fallah S, Shephard RJ (2008). Usefulness of improvement in walking distance versus peak oxygen uptake in predicting prognosis after myocardial infarction and/or coronary artery bypass grafting in men. Am J Cardiol.

[25] Grazzi G, Myers J, Bernardi E, Terranova F, Grossi G, Codeca L, Volpato S, Conconi F, Mazzoni G, Chiaranda G (2014). Association between VO(2) peak estimated by a 1-km treadmill walk and mortality. A 10-year follow-up study in patients with cardiovascular disease. Int J Cardiol.

[26] Epstein M (1976). Cardiovascular and renal effects of head-out water immersion in man: application of the model in the assessment of volume homeostasis. Circ Res.

[27] Buemi M, Corica F, Di Pasquale G, Aloisi C, Sofi M, Casuscelli T, Floccari F, Senatore M, Corsonello A, Frisina N (2000). Water immersion increases urinary excretion of aquaporin-2 in healthy humans. Nephron.

[28] Farrow S, Banta G, Schallhorn S, May R, Mers A, Cadaret L, Rydstedt L, Lockette W (1992). Vasopressin inhibits diuresis induced by water immersion in humans. Journal of applied physiology (Bethesda, Md : 1985).

[29] Broman G, Quintana M, Engardt M, Gullstrand L, Jansson E, Kaijser L (2006). Older women's cardiovascular responses to deep-water running. J Aging Phys Act.

[30] Sheldahl LM, Tristani FE, Clifford PS, Hughes CV, Sobocinski KA, Morris RD (1987). Effect of head-out water immersion on cardiorespiratory response to dynamic exercise. J Am Coll Cardiol.

[31] Gerbes AL, Arendt RM, Gerzer R, Schnizer W, Jungst D, Paumgartner G, Wernze H (1988). Role of atrial natriuretic factor, cyclic GMP and the renin-aldosterone system in acute volume regulation of healthy human subjects. Eur J Clin Investig.

[32] Zanettini R, Bettega D, Agostoni O, Ballestra B, del Rosso G, di Michele R, Mannucci PM (1997). Exercise training in mild hypertension: effects on blood pressure, left ventricular mass and coagulation factor VII and fibrinogen. Cardiology.

[33] De Meersman RE (1993). Heart rate variability and aerobic fitness. Am Heart J.

[34] Takeshima N, Rogers ME, Islam MM, Yamauchi T, Watanabe E, Okada A (2004). Effect of concurrent aerobic and resistance circuit exercise training on fitness in older adults. Eur J Appl Physiol.

[35] Colado JC, Triplett NT, Tella V, Saucedo P, Abellan J (2009). Effects of aquatic resistance training on health and fitness in postmenopausal women. Eur J Appl Physiol.

[36] Fonong T, Toth MJ, Ades PA, Katzel LI, Calles-Escandon J, Poehlman ET (1996). Relationship between physical activity and HDL-cholesterol in healthy older men and women: a cross-sectional and exercise intervention study. Atherosclerosis.

[37] Goodyear LJ, Hirshman MF, Horton ES (1991). Exercise-induced translocation of skeletal muscle glucose transporters. Am J Phys.

[38] Nowak A, Pilaczynska-Szczesniak L, Sliwicka E, Deskur-Smielecka E, Karolkiewicz J, Piechowiak A (2008). Insulin resistance and glucose tolerance in obese women: the effects of a recreational training program. J Sports Med Phys Fitness.

[39] Rolland YM, Cesari M, Miller ME, Penninx BW, Atkinson HH, Pahor M (2004). Reliability of the 400-m usual-pace walk test as an assessment of mobility limitation in older adults. J Am Geriatr Soc.

